# Vineyard Yield Estimation Based on the Analysis of High Resolution Images Obtained with Artificial Illumination at Night

**DOI:** 10.3390/s150408284

**Published:** 2015-04-09

**Authors:** Davinia Font, Marcel Tresanchez, Dani Martínez, Javier Moreno, Eduard Clotet, Jordi Palacín

**Affiliations:** Department of Computer Science and Industrial Engineering, Universitat de Lleida, Jaume II, 69, 25001 Lleida, Spain; E-Mails: dfont@diei.udl.cat (D.F.); mtresanchez@diei.udl.cat (M.T.); dmartinez@diei.udl.cat (D.M.); jmoreno@diei.udl.cat (J.M.); eclotet@diei.udl.cat (E.C.)

**Keywords:** precision agriculture, yield estimation, segmentation techniques, color features

## Abstract

This paper presents a method for vineyard yield estimation based on the analysis of high-resolution images obtained with artificial illumination at night. First, this paper assesses different pixel-based segmentation methods in order to detect reddish grapes: threshold based, Mahalanobis distance, Bayesian classifier, linear color model segmentation and histogram segmentation, in order to obtain the best estimation of the area of the clusters of grapes in this illumination conditions. The color spaces tested were the original RGB and the Hue-Saturation-Value (HSV). The best segmentation method in the case of a non-occluded reddish table-grape variety was the threshold segmentation applied to the H layer, with an estimation error in the area of 13.55%, improved up to 10.01% by morphological filtering. Secondly, after segmentation, two procedures for yield estimation based on a previous calibration procedure have been proposed: (1) the number of pixels corresponding to a cluster of grapes is computed and converted directly into a yield estimate; and (2) the area of a cluster of grapes is converted into a volume by means of a solid of revolution, and this volume is converted into a yield estimate; the yield errors obtained were 16% and −17%, respectively.

## 1. Introduction

One of the most important food industries is the grape growing and wine-making industry, which is currently introducing several enhanced vineyard management techniques, which involve automatic leaf estimation [1], fruit harvesting [2,3], yield estimation [4,5,6,7], grape quality evaluation [8] and grapevine variety identification [9]. The wine industry has the challenges of performing accurate yield prediction, estimation and quality control [10], because such factors are affected by environmental variables (soil factors, climate, plant diseases), forecast and pollen concentration [11], farming factors, such as adding products (water, pesticide, fertilizer, herbicides) [12,13], and agricultural tasks [14] (number of sprouts, informed pruning, shoot thinning, bunch thinning, number of bunches, prune weight, *etc.*), which makes the feasible management of the vineyard more complicated.

Crop management can be improved by using remote sensors [6] configured in airborne [14,15] and terrestrial applications, such as crop classification, crop area estimation, canopy measurements, identification of harvest dates, crop yield estimation, detection of pest occurrence, detection of disease occurrence, mapping weed infestation and monitoring abiotic stress. For example, in [7], a terrestrial LIDAR device was proposed in order to obtain canopy volume and tree shape information in peach orchards and to analyze relationships between the measured LIDAR tree volume and yield and fruit weight. The conclusion obtained was that the LIDAR is a suitable technique to assess fruit tree production capacity. Another alternative is the use of specialized terrestrial vision systems. Another example is the location and detection of fruits on trees [16] by placing a camera at different positions and different viewing angles (azimuth and zenith angles). In this case, the best results were obtained when locating the camera in front of the fruit with a zenith angle of 60° upwards. Additionally, the maximum detection of fruit (90%) was achieved when using five multiple viewpoints positions. In [17], a new method based on segmenting the point cloud obtained by using a 3D camera into convex surfaces was implemented for individual fruit recognition and detection. The conclusions obtained were that the proposed method can be used for fruit detection, although this detection is extremely sensitive to changes in lighting conditions [4,5,18] and the color similarity between the fruit and the background [19]. In this direction, in [20], an image-processing technique was proposed to detect fruits of different degrees of ripeness by using RGB images in combination with automatic machine learning, obtaining classification ratios from 0.78 to 1.00 for different ripening conditions. In [21], the specific problems originating from daylight operation were identified: skylight transmission from the back side of trees, direct sunlight reflectance from non-fruit materials and variations in natural lighting. Similarly, in [22], it was proven that changing solar angles, cloud cover and leaf occlusions leads to lighting variations that complicate the segmentation process.

In order to manage the fruit skin color variability in images, the proposals of [18,23] were to address fruit skin color daylight variability by defining a linear color model in the RGB color space and computing the pixel color intensity distance to these models for direct fruit segmentation. Nevertheless, the general conclusion is that the same segmentation techniques cannot be applied to different scenarios [24].

Regarding the specific case of a vineyard, the problem is the definition of an automatic procedure to recognize and identify grapes or clusters of grapes [19] in order to estimate yield or the optimal harvesting period. In general, this detection is affected by color similarities between grapes and background, the different size and scale of the grapes, occlusion originating from leaves or/and branches, weather conditions, light variations and reflections and shadows. For example, the proposal of [5] was to characterize the diameter and the ripeness of grapes in vineyard images. The segmentation process was performed in the HSI (hue, saturation and intensity) color space by applying a threshold segmentation level and two additional restrictions: the image regions must have a predefined range of intensities and the objects must have a feasible diameter. In [25], the proposal to avoid illumination variability was to perform the grape segmentation procedure in the CIELab color space. This segmentation considers the lightness component of the pixels in relation to the color characteristics of the crop allowing a segmentation quality of 87.2%. In [26], an automatic system to detect berries and to determine their size and weight was proposed. In this case, the segmentation technique was based on applying a Bayesian discriminant model by using as inputs the RGB pixels from two classes of objects selected in the image; background and fruit (peel/stem). Then, these results were stored in a look up table (LUT) to perform a fast segmentation. The system estimates the berry weight (R2 > 0.96) and size (R2 > 0.97) properly, extending the suitability of the system to other types of fruits and vegetables.

The use of artificial illumination at night was proposed in [27], where color mapping combined with morphological operators was used to localize clusters of red and white grapes, obtaining a cluster detection efficiency of 97% and 91%, respectively. More recently, [28] showed the first complete system for vineyard yield estimation, which has been evaluated with artificial illumination at night over several years and a large quantity of vines of different vineyards. In this case, the system captures 75% of the spatial yield variance with average errors between 3% and 11% of the total yield, values that represents the state-of-the-art in this field.

In a similar direction, the proposal of this paper is to perform yield estimation by applying controlled artificial illumination at night in a vineyard in order to avoid the color variability and changes induced by daylight natural illumination. The main goal is to assess different methods suitable for grape segmentation in vineyard images, perform an estimate of the area and volume of the cluster of grapes and, finally, estimate the vineyard yield.

## 2. Materials and Methods

The materials used in this paper are the vineyard facility and the image acquisition system. The methods used are different image processing techniques, which will be later optimized in order to properly segment the area of the clusters of grapes in the vineyard images.

### 2.1. Vineyard Facility

The vineyard facility was located in Bakersfield, California. The grapes produced in this vineyard correspond to the red table-grape Flame Seedless grape variety. These grapes are usually ripe in July and are characterized for their small size, round shape, firm and crisp reddish texture and seedless property [29]. In the case of this high-quality table-grape variety, an accurate yield estimate will contribute to optimizing vineyard management as a way to reduce production costs. The different segmentation procedures assessed later in this paper will be optimized to detect this reddish table-grape variety. The detection of other grape varieties will require the repetition of the assessment.

### 2.2. Image Acquisition System

The image acquisition system was composed of a high-resolution monocular Nikon D300s camera (Nikon Inc., Melville, NY, USA) with Nikon AF-S DX Nikkor 18–55-mm 1:3.5–5.6 G lenses and a focal length of 20 mm. This camera offers many possibilities for remote control and external image triggering. In this paper, the aperture of the camera was set to F11 with ISO200; the exposition time was set to 1/250 s; and no exposure compensation was applied. The images acquired were saved as RGB jpeg images with 4288 × 2848 pixels, 24-bit color depth and a file size smaller than 6 MB. The artificial illumination was generated mainly with a ring flash mounted over the camera with the energy released configured to 10 Ws. This ring illuminator provides a bright and uniform illumination in the complete area of the images acquired. The complete illumination system was pointed 45° to the ceiling and mounted in an auxiliary utility vehicle in order to explore the vineyard and obtain high-resolution and high-quality images of high-quality red-table grapes during the night.

**Figure 1:**
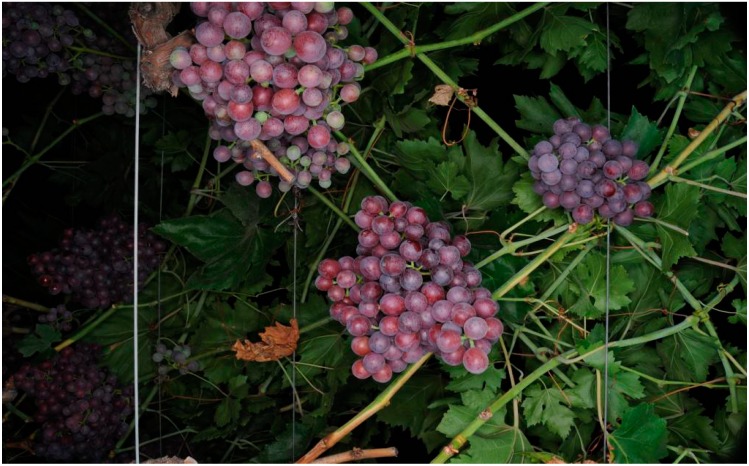
Example vineyard image: 4288 × 2844 pixels.

The utility vehicle was driven by an expert human operator at a constant speed and at an approximate distance of 2 m from the vines of both sides. The vineyard images were sampled at a fixed interval assuming no overlap between consecutive images. Under such conditions, the average diameter of a mature individual red grape is 109 pixels and the average area of a cluster of grapes is 250,000 pixels. Figure 1 shows a typical vineyard image. The hypothesis is that the use of high-resolution and high-quality images in controlled illumination conditions during the night will simplify the detection of individual clusters of grapes, simplifying the estimation of the areas of the clusters and improving the vineyard yield estimate.

The vineyard images analyzed in this paper were obtained during the harvesting period. The real-time analysis of the acquired images and the synchronization between the trigger applied to the camera and the displacement of the utility vehicle will be addressed in future works.

## 3. Yield Calibration

The objective of the yield calibration stage is the development of a procedure designed to obtain the relationship between grape-cluster image analysis parameters (measured directly in pixels) and grape-cluster weight (measured in grams). The calibration parameters selected in this paper are the area of the cluster of grapes and the volume of the cluster of grapes, but if available, other parameters, such as the number of grapes in the images, can be used as calibration parameters [28,30,31]. These calibration parameters will enable the automatic estimate of the vineyard yield based on the analysis of the images obtained from the vineyard. The typical hypotheses adopted in this calibration procedure are:

- There is a relationship between the weight, size and volume of the cluster of grapes [4,10,28,30,31].
- The obtained relationship between the weight and size of the cluster of grapes is valid during a measurement experiment.
- The grape variety analyzed in this paper is of high quality and low cluster density.
- The distance between the grapes and the image acquisition system is constant during the entire process [28,32].

**Figure 2:**
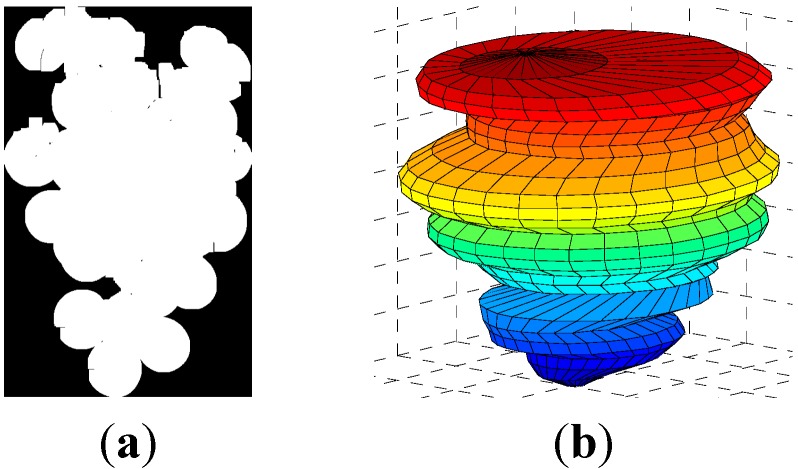
(**a**) Example segmented image of a cluster of grapes; (**b**) Representation of the solid of revolution of the cluster of grapes estimated from the segmented image.

Similarly to the calibration procedure proposed in [28], the proposal for yield calibration has been performed off-line in laboratory conditions in order to guarantee enough weight and cluster size variability, but a practical development of this proposal will require the development of a detailed on-line application procedure. The yield calibration procedure is based on the following steps: (1) a set of representative clusters of grapes were manually harvested and selected for the calibration; (2) the weight of each cluster of grapes is manually measured; (3) each cluster of grapes is hung in front of a white background in order to obtain a reference image with the image acquisition device; (4) the image of the cluster of grapes is automatically segmented by applying the Otsu method [33] combined with the application of morphological operators (10 erosions and dilations) in order to remove noisy pixels from the images (Figure 2a); and (5) the segmented image is used to estimate the area and volume of the cluster of grapes expressed in pixels. The total area of the cluster of grapes is computed as the number of white pixels in the segmented image (Figure 2a). This area estimate summarizes the effect of all of the existing grapes in a cluster. Alternatively, the proposal is to compute the volume of the clusters. For example, in [28], the volume of the clusters is estimated by using a 3D ellipsoidal model, but in this paper, the proposal is to estimate the volume of the grapes by interpreting the area of the grapes as the volume of a solid of revolution (expressed in pixels or square pixels). This volume estimate is computed similarly as a solid of revolution (Figure 2b), where the cylinder of each row is obtained around the column center of the object. For example, if *k* is a row of the segmented image and *s(k)* and *e(k)* the first and last image column of the pixels classified as a cluster of grapes, then this partial volume slice will be computed as *π·((e(k) − s(k))/2)^2^*.

**Figure 3:**
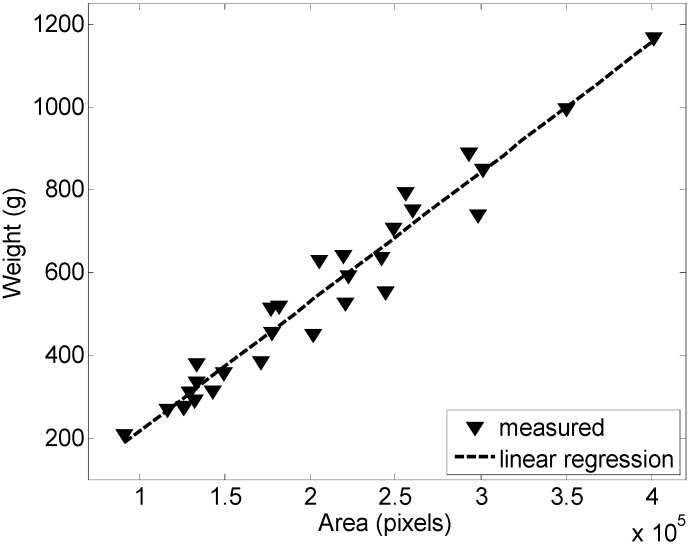
Relationship between the weight and area of the cluster of grapes analyzed.

**Figure 4:**
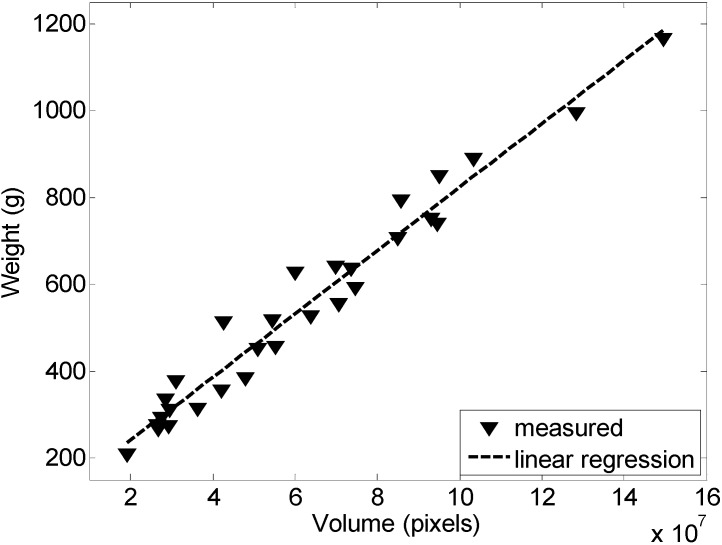
Relationship between the weight and volume of the cluster of grapes analyzed.

Figure 3 and Figure 4 show the calibration results obtained with 29 representative clusters of grapes. Figure 3 shows the relationship between the areas of the cluster of grapes (expressed in pixels) and its weight (in grams); the coefficient of correlation was 0.9557. Alternatively, Figure 4 shows the linear relationship between the volume of the cluster of grapes (expressed in pixels) and its weight (in grams); the coefficient of correlation was 0.9635. In [31], similar results have been achieved in a less controlled environment.

## 4. Automatic Segmentation of Clusters of Grapes

This section is focused on the automatic segmentation of the cluster of grapes as a procedure required to perform an estimate of the yield. The hypothesis is that the controlled artificial illumination used during the acquisition of the images at night will simplify the grape segmentation procedure and will allow the application of a pixel-based segmentation method. The main advantage of using a pixel-based classification strategy is that complex classification rules can be mapped directly into a LUT [23] and applied in a real-time implementation.

The pixel-based segmentation procedures assessed in this section are: threshold segmentation, Mahalanobis distance segmentation, Bayesian classifier, direct three-dimensional histogram and linear color models. The empirical assessment is based on the analysis of a continuous sequence of vineyard images acquired with the image acquisition system under controlled artificial illumination at night. In this paper, the images of the cluster of grapes analyzed are not affected by occlusion. The final goal of this proposal is to classify all of the pixels of the high-resolution vineyard images into grapes (“1” or white color) or background (“0” or black color). The color spaces analyzed were the original RGB and the Hue-Saturation-Value (HSV) with the H layer shifted 180° in order to move the reddish components of the grapes into the center of the H linear vector.

**Figure 5:**
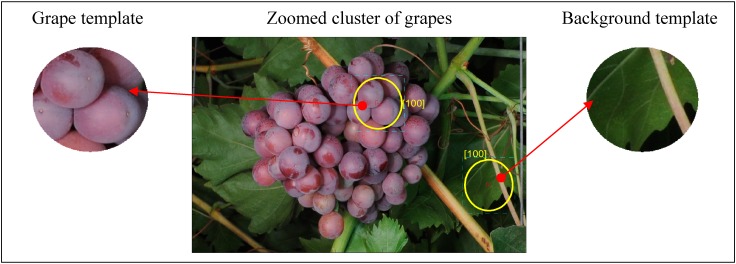
Example of a manual selection of the grape and background templates.

The tuning of the segmentation procedures used in this paper may require the previous manual selection of complementary reference templates in a representative image of the vineyard. In this paper, the proposal is to define two basic auxiliary templates (Figure 5): grape template and background template. In the case of the grape template, a predominant reddish color from the skin of the grapes is expected, but in the case of the background template, a mixture of different predominant colors from leaves, branches and other dark image areas is expected. In this paper, these two templates will be required by some segmentation methods in order to define two basic classification classes: template and background. The effect of defining more classes (for example, splitting the background class into leaves, branches and dark background classes) has not been evaluated, except in the case of using linear color models, because doing so is mandatory for this method. Figure 5 shows a zoomed part of a representative vineyard image where an expert human operator has already selected two reference templates by applying a circular selection tool.

**Figure 6:**
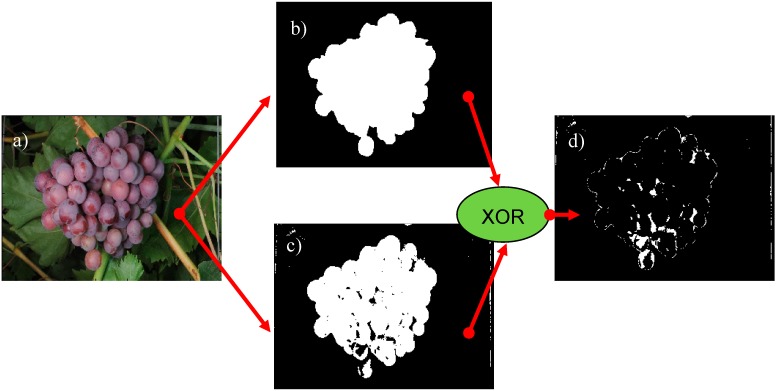
(**a**) Vineyard image with a cluster of grapes; (**b**) Manual labeling of the cluster of grapes; (**c**) Example automatic grape segmentation results; (**d**) XOR differences between the manual labeling and the automatic segmentation.

The accurate validation of the different cluster grape segmentation methods assessed requires two operations: (1) an accurate manual labeling of the regions of the images covered by grapes; and (2) the comparison of the automatic classification results with the manual labeling in order to extract statistical similitude information. As an example, Figure 6 shows this validation procedure applied to one cluster of grapes of the vineyard (Figure 6a). Then, Figure 6b shows the accurate result obtained with a manual selection of the contour of the cluster of grapes (with approximately 600 contour points), and Figure 6c shows an example of automatic pixel-based grape segmentation. Finally, Figure 6d shows the differences between the manual labeling of the cluster and the automatic segmentation by applying an exclusive (or XOR) function between both segmented images. Finally, the size segmentation error is computed in this paper by counting the different pixels (the XOR differences) divided by the number of pixels of the cluster of grapes obtained from the manual selection of the contour. These image differences can be further reduced by applying an optimized sequence of morphological operators to the segmented image.

The color spaces considered for all of the methods assessed in this paper were the original RGB color space and the transformed HSV color space [34], with the hue component shifted 180° in order to place the red color intensity in the center of the H plane and to simplify the detection of the predominant reddish color of the grapes.

The segmentation results obtained with each method assessed can be improved with the application of an empirical optimized sequence (or minimal sequence) of morphological operators, such as the hole filling, erosion and dilation, in order to refine the segmentation. This empirically-optimized morphological filtering sequence must be interpreted as the optimal or fast combination of erosion and dilatation operators required to eliminate noisy pixels from the images. In the case of big objects, such as the cluster of grapes, the use of a larger sequence with more erosion and more dilation operators only results in requiring greater execution time, because both operators have a neutral compensating effect.

### 4.1. Threshold Segmentation

The application of threshold levels in one or several color layers in order to segment the areas covered by grapes or other fruits in color images is very common in the literature. For instance, in [35], a transform to the original RGB color space into the YIQ color space was proposed, and the threshold intensity levels were established by a trial and error manual operation. The proposal of [36] was to implement one of the methods for vineyard yield estimation by establishing the red, green and blue thresholds in a reference RGB image manually and to apply these settings to the remaining images. In [37], a transform from the RGB image into the Ohta color space [38] was proposed by applying a constant threshold for fruit segmentation. In [5], the grapes were segmented by applying a threshold level to the transformed HSI color space [39]. The threshold levels were computed by analyzing the histogram of the transformed images [40]. Similarly, in [27], the grapes in vineyard images taken at night were detected by defining a region of pixel intensities in the RGB color space to segment the grapes based on a trial and error procedure.

Inspired by these cited works, the threshold segmentation method is applied to five different cases: red (R), green (G), blue (B), gray (I) and transformed hue (H) layers, in order to take advantage of the existing color differences between the reddish grapes and the greenish background. In each intensity color layer considered, the segmentation threshold level was fixed by applying the Otsu method [33] to the first image acquired with the image acquisition device.

### 4.2. Mahalanobis Distance Segmentation

Mahalanobis distance [41] segmentation is the second method tested, which consist of computing the distance between the three-dimensional color intensities of the pixels of the image and the ellipsoidal pixel intensity distributions of the existing grape and background templates. This distance can be used to classify each pixel as a member of its closest template. Compared with the Euclidean distance, the Mahalanobis distance also considers the differences in the variances of each intensity layer of the reference templates, so it is a robust method against small color variance caused by small changes in lighting. For example, in [4], eight different templates were proposed in daylight field conditions: grapes, wood, background and four classes of leaf, depending on their age.

Inspired by these cited works, the Mahalanobis distance segmentation will be applied with only two classes: grape and background. The variances of each intensity color layer were obtained by analyzing the grape template and the background template (selected in the first image obtained in the vineyard). Then, the color intensity of an image pixel is compared with those models by computing the Mahalanobis distance, and the pixel is classified with the nearest class.

### 4.3. Bayesian Classifier

The Bayesian classifier is a well-known classifier widely used for image analysis in agricultural applications. For instance, in [42], a Bayesian classifier to detect plants was implemented, and in [43], oranges in trees were discriminated with the aim of providing guidance information for a robotic manipulator.

The Bayesian classifier is a probabilistic technique based on the previous definition and analysis of image features (pixel color intensities in this paper) corresponding to different classes. Based on these characteristic statistical features, a Bayesian classifier is able to analyze and classify each pixel of the image into one of these specified classes. Inspired by the work of [43], the implementation of the Bayesian classifier is based on the simplified discriminant function, which assumes that the covariance matrices of the two reference templates used in the learning stage are not equal, and the color features that describe the grapes and the background are not statistically independent. Finally, the color intensity of a pixel is classified as a member of the template class with a large discriminant value.

### 4.4. Linear Color Model Segmentation

The proposal of applying linear color models (LCM) to detect fruit in color images [18] is based on the prior selection of small object region areas in the image whose pixels have a linear intensity relationship (in a three-dimensional space) that can be modeled with a linear regression. Then, a class or object is defined by several linear regressions that describe the different color relationships of the object, and the pixel color intensity is classified by finding the minimum Euclidean distance to all the linear regressions defined. This classification method is very powerful, as it can model objects with a non-uniform color distribution and affected by daylight illumination changes, but requires the definition of one class per image object and several templates per class in order to model all object color variabilities. In this paper, three different template selections have been used to define the color variability of the grape class, and six different template selections (leaves, branches, *etc.*) have been used to define the color variability of the background (or non-grape) class.

### 4.5. Histogram Segmentation

An alternative way to detect the skin of the grapes in the images is by comparing pixel color intensities with an existing three-dimensional color-intensity histogram obtained from a grape template (either in the RGB and HSV color spaces). However, this detection method is dependent on the manually selected grape template and may require the selection of additional grape templates in order to include all of the skin-color relationships of the grapes. In order to overcome this problem, the proposal of [27] was to fill the gaps in the three-dimensional histogram and to complete the color relationship by applying a morphological dilation with a structuring element of 3 × 3 boxes. Inspired by this proposal, this paper proposes to dilate the color relationships appearing in the three-dimensional color-intensity histogram computed from the grape template by convolving the histogram with a solid sphere. Then, the segmentation is performed with a zero threshold level applied to the histogram.

## 5. Results

### 5.1. Grape Cluster Size Segmentation Results

This subsection presents the pixel-based segmentation results obtained. Table 1 enumerates the segmentation method, the color space analyzed, the values of the tunable parameters of each method, the clusters detected, the cluster size estimate error after segmentation (Segm. in Table 1), and the improvement obtained when applying a morphological filtering (Seg. + Morph. Filter in Table 1) for noise removal and the optimal (or minimum) morphological filtering sequence applied, obtained by a trial and error procedure in one sample image. The results of Table 1 have been obtained by analyzing 97 clusters of grapes obtained from 40 vineyard images (see Figure 1). This proposal agrees with [28], where a small amount of labeled images was used to identify the most optimal grape descriptor. In this paper, all of the methods assessed are color dependent, so the optimal results are tuned for the mature reddish grape variety analyzed in this paper; it is expected that the segmentation of damaged grapes, grape varieties with other characteristic skin color or even the same variety, but in another maturity state, may produce different results.

In general, Table 1 shows that the detection of non-occluded clusters of grapes is always successful, probably because the skin color variability of the grapes is limited by the use of controlled illumination at night. Then, the quality of the segmentation procedure can be evaluated in a fine way by computing the number of different pixels (false positives and false negatives) between the automatic segmentation provided and the manual labeling of the clusters.

The first method (first bloc of rows) illustrated in Table 1 corresponds to the threshold segmentation method applied to different color spaces. In this case, the best segmentation results and the best cluster size estimate were obtained when applying a threshold (0.54902) to the H layer, obtaining an error of 13.55%, improved to 10.01% by applying a morphological filtering sequence (a hole filling (HF) combined with four erosions (4E) and four dilations (4D)). The next method shown is the Mahalanobis segmentation (Mah. Segm. in Table 1) in the RGB and HSV color spaces. The use of the Mahalanobis distance has the advantage that it does not require additional configurable or segmentation parameters, as it only requires the definition of reference templates [4], the determination of the Mahalanobis distance, and the classification of the pixels according to the class of the nearest Mahalanobis class. Table 1 shows that the average size error obtained was 17.36% in the RGB color space and 16.05% in the HSV color space, and these values were improved up to 13.29% and 10.50% by the application of an optimal sequence of morphological operators: four erosions (4E), four dilations (4D) and a hole filling (HF); and three erosions (3E), three dilations (3D) and a hole filling (HF), respectively. The cluster size estimate errors obtained with the Bayesian segmentation (Bay. Segm. in Table 1) in the RGB and HSV color spaces are similar (19.29% and 17.97%). The development of the Bayesian classifier requires the previous definition of the baseline probability for the two classes used: grapes and background. In this paper, the assumption made is that all of the pixels of the image must be members either of the grape or background class, and then, the best separation between both classes was with a pixel prior probability of 43.36% for the grape class and 56.64% for the background class, values obtained by a trial and error procedure during the tuning stage. The errors obtained with the Bayesian classifier were improved up to 13.24% and 10.29% with the application of an optimal sequence of morphological operators: five erosions (5E), five dilations (5D) and a hole filling (HF); and three erosions (3E), four dilations (4D) and a hole filling (HF), respectively. The next bloc of rows show the results obtained with the LCM segmentation, which provides better cluster size estimations results in the RGB color space (20.07% error) rather than the HSV (62.59% error), although the application of a sequence of morphological operators greatly reduces the cluster size estimation error up to 10.99% and 14.78%, respectively. The optimal sequence of morphological operators applied was: five dilations (5D), five erosions (5E) and a hole filling (HF) in the RGB color space; and three dilations (3D), four erosions (4E) and a hole filling (HF) in the HSV color space. The LCM segmentation method was originally proposed to model color relationships in the RGB color space, and it is expected to have the best segmentation performances in this color space. The drawback of the LCM method is the accurate manual selection of several representative templates during the tuning stage in order to describe the color variability of grapes and the other objects considered as part of the background. Finally, the histogram segmentation (Hist. Segm. in Table 1) technique generates similar segmentation results and similar cluster size estimations in the RGB color space (18.80%, improved up to 13.37% when applying morphological operators) and in the HSV color space (17.81%, improved up to 12.27%). In both cases, the resulting three-dimensional histogram was computed with 128 histogram bins per layer, dilated with a solid sphere with a radius, r, of six bins in the RGB color space and nine bins in the case of the HSV color space. These radii were obtained by a trial and error procedure during the tuning stage.

**Table 1:** Grape cluster size segmentation results obtained in the case of no occlusion.

				Average Grape Cluster Area Error		
				(40 Images/97 Clusters of Grapes)		
Method	Color Space	Parameters	Clusters Detected	Segm.	Segm. + Morph. Filter	Morphological Filter *
**Threshold Segmentation**	R	R > 0.35294	100%	59.67%	62.54%	6E + 4D + HF
	G	G > 0.29412	100%	87.87%	95.61%	8E + 2D + HF
	B	B > 0.29804	100%	57.00%	55.00%	4E + 6D + HF
	Gray	I > 0.30588	100%	74.06%	80.00%	7E + 3D + HF
	H	H > 0.54902	100%	**13.55%**	**10.01%**	HF + 4E + 4D
**Mah. Segm.**	RGB	-	100%	17.36%	13.29%	4E + 4D + HF
	HSV	-	100%	16.05%	10.50%	3E + 3D + HF
**Bay. Segm.**	RGB	43.36%/56.64%	100%	19.29%	13.24%	5D + 5E + HF
	HSV	43.36%/56.64%	100%	17.97%	10.29%	3D + 4E + HF
**LCM Segm.**	RGB	-	100%	20.07%	10.99%	5D + 5E + HF
	HSV	-	100%	62.59%	14.78%	3D + 4E + HF
**Hist. Segm.**	RGB	*r* = 6	100%	18.80%	13.37%	3E + 3D + HF
	HSV	*r* = 9	100%	17.81%	12.27%	3E + 3D + HF

* Morphological operators: E, erosion; D, dilation; HF, hole filling.

In general, small differences in the numerical errors obtained with the methods compared have a large impact on the accuracy of the segmentation, because the large size of the images analyzed smoothes the differences in the perimeter of the cluster of grapes. The lowest difference between the areas of the cluster of grapes (automatic segmentation compared to manual labeling) was 13.55% in the case of applying a threshold level to the H color layer, and this error was improved up to 10.01% when applying an optimal sequence of morphological operators. This segmentation method provides the best estimate of the area (less false negatives and false positives) and has the advantage that it is extremely fast and then suitable for a real-time implementation, as it only requires the computation of the transformed H color layer of the image and the application of a threshold level.

Alternatively, in the case of using the original RGB color space, the lowest size estimation error was obtained when applying the linear color model segmentation (10.99%), although this method has the drawback of requiring an accurate selection of several representative templates of the grapes, branches, leaves and shadows appearing in the images. This initial selection is reduced to only two templates in the case of the Bayesian classification (13.24% error), the Mahalanobis distance segmentation (13.29% error) and the histogram segmentation (13.37% error). This initial selection precludes the practical applicability of these cited segmentation methods.

The segmentation results obtained agree with the classification results obtained by [27] and validate the use of controlled artificial illumination at night for grape detection as it generates small light/brightness variations in the skin of the grapes and simplifies fruit segmentation.

### 5.2. Yield Estimation Results

**Figure 7:**
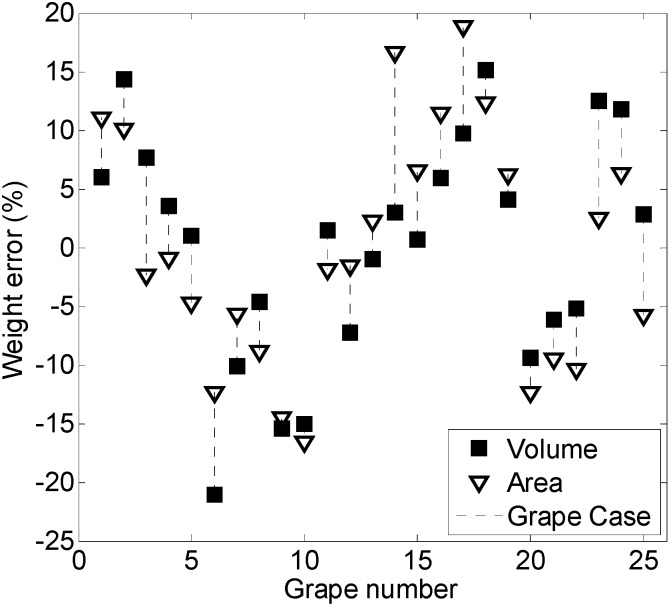
Individual error obtained when comparing the real and estimated weight of 25 clusters of grapes in consecutive images. The grape weight was predicted by using the estimated area and deduced volume of the cluster of grapes.

Finally, the area of the clusters of grapes obtained with the best segmentation method (applying a threshold to the H color layer) can be converted into a yield estimate by applying the regression curves obtained in the calibration stage. Figure 7 shows the error obtained when estimating the individual weight of a sequence of 25 clusters of grapes computed from the volume and area of the pixels of the grapes; with individual error values from 15.1% to −21.1% and 18.8% to −16.5%, respectively.

The cluster of grapes shown in Figure 7 is not affected by occlusion, because the hypothesis is that the table-grape variety is properly trimmed for a fresh marker production. However, any occlusion in the grapes will reduce the area of the grapes in the images, reduce the volume of the solid of revolution and then reduce the weight estimated with both methods.

Finally, Table 2 shows the total weight estimate and the average error produced for the cases of using the grape cluster area and the grape cluster volume obtained from the images analyzed. In the case of estimating the weight from the area of the cluster of grapes, the total weight computed for 25 non-occluded clusters was 18.382 kg, which represents an error of 16% relative to the real weight measured. Alternatively, in the case of performing this estimate from the estimated volume of the cluster of grapes; the total weight computed for these 25 non-occluded clusters was 13.183 kg, which represents an error of −17% relative to the real weight measured. Table 2 also shows that the average estimations obtained with both methods can be compensated, although this effect can be a numerical coincidence, and future extensive validation experiments performed with large datasets will be needed to validate this compensation effect.

The calibration results obtained in Section 3 showed a linear relationship between the weight and the area of the clusters and between the weight and the deduced volume of the clusters (obtained as a solid of revolution of the area of the grapes), but the yield error results obtained in this experimental measurement (16%, −17%) are not conclusive of which method is most suitable for this estimation. Initially, the expectation was to obtain better yield estimates when using the volume of the grapes (obtained as a solid of revolution from the segmented area), but the results have not confirmed this expectation, and other, similar proposals also concluded that a volume estimate will be very sensitive to cluster overlapping and occlusion [28]. The relative yield error obtained when segmenting the vineyard images with the other methods assessed in this paper is worse than the results shown in Figure 7 and Table 2, because the calibration curves are very sensitive to the estimated cluster size; a higher error in the size estimate after segmentation is converted into a higher yield estimate error.

**Table 2:** Total yield estimate for the case of 25 clusters of grapes.

Weight Estimated From	Total Estimated Weight (kg)	Total Measured Weight (kg)	Error (%)
Grape cluster area	18.382	15.835	16.0
Grape cluster volume	13.183	15.835	−16.7
Average	15.782	15.835	−0.3

The final vineyard yield error obtained with the two methods proposed in this paper in the case of analyzing a limited number of clusters has a similar error range as similar works found in the literature. In [28], the yield error obtained in a realistic experimentation with a controlled illumination was between 3% and 11% when evaluating the information corresponding to several years, hundreds of vines and four grape varieties. In [44], the absolute yield errors reported were in a range between 9 and 15% when using a contact-based estimator and historical data, whereas in [30], the yield error reported in a row of vines was of 9.8%. In the case of other fruits, a yield error within a 10% range is considered valuable in terms of crop management [32].

Finally, the methods proposed in this paper require further validation by performing large vineyard measurements, such as presented in [28], although such a task was beyond the scope and possibilities of the present paper.

## 6. Conclusions

This paper proposed a method for vineyard yield estimation based on the application of artificial illumination in a vineyard facility during the night in order to obtain high-resolution images of clusters of red grapes with small illumination variance. Five grape segmentation methods have been empirically assessed under such illumination conditions. The quality of the segmentation was computed by comparing the automatic segmentation results with a manual labeling of the clusters of grapes in the images. The direct segmentation results have been improved by applying a sequence of morphological operators in order to fill gaps and eliminate noisy pixels from the segmented images.

Empirical results showed that the use controlled illumination at night combined with the high-resolution and high-quality images of the vineyard simplifies the detection of clusters of grapes, because the color variability is small and the number of pixels available in each grape is very large (250,000 pixels on average). In the case of clusters of grapes not affected by occlusion, nine optimized implementations have provided grape size errors under 15%. The best estimate of the area of the grapes was obtained when applying a threshold level to the transformed H color layer of the images, obtaining a cluster size estimate error of 13.55%, which was improved up to 10.01% when applying an optimized sequence of morphological operators. In this case, the H layer was shifted 180° in order to move the reddish components of the grapes into the center of the H circular vector. The additional advantage of threshold segmentation is the simplicity of its real-time implementation in an agricultural machine designed to locate clusters of grapes and to estimate their size.

The area and estimated volume of the clusters of grapes obtained from the images analyzed have been converted to vineyard yield by using specific calibration curves, which require manual operation. The results obtained with the proposed methods have shown that the yield can be predicted with an error of 16% and −17% in the cases of using the size information of the segmented area of the grapes and the volume of the solid of revolution computed from the segmented area.

The results of this paper will have application to vineyard management by optimizing the resources required to harvest, transport, store and, if needed, manufacture the vineyard product. Future work will be focused on developing a real-time system in order to estimate yield in large vineyard facilities and to evaluate the robustness of the estimators against diseases and the ripening stage of the grapes.
